# Provision of COVID-19 Self-Test Kits to Patients for Distribution to Social Contacts: A Randomized Clinical Trial

**DOI:** 10.1001/jamanetworkopen.2025.13708

**Published:** 2025-06-04

**Authors:** Cedric H. Bien-Gund, Alisa J. Stephens-Shields, Trisha Acri, Karen Dugosh, Robert Gross

**Affiliations:** 1 Division of Infectious Diseases, Department of Medicine, University of Pennsylvania Perelman School of Medicine, Philadelphia; 2 Leonard Davis Institute of Health Economics, University of Pennsylvania, Philadelphia; 3 Department of Biostatistics, Epidemiology, and Informatics, University of Pennsylvania Perelman School of Medicine, Philadelphia; 4 Courage Medicine Health Center, Philadelphia, Pennsylvania; 5 Public Health Management Corporation, Philadelphia, Pennsylvania

## Abstract

**Importance:**

Widespread and equitable access to testing remains critical to controlling the COVID-19 pandemic, which has disproportionately affected medically underserved communities.

**Objective:**

To determine whether secondary distribution of COVID-19 self-test (ST) kits, in which an individual distributes ST kits to contacts in their social networks, increases COVID-19 testing.

**Design, Setting, and Participants:**

The COVID-19 Self-Testing Through Rapid Network Distribution study was a randomized clinical trial conducted between May 2021 and September 2023 at 4 federally qualified health centers serving medically underserved populations in Philadelphia, Pennsylvania. Participants were adults aged 18 years or older presenting to federally qualified health centers without SARS-CoV-2 infection in the past 90 days. Participants were randomized 1:1 to receive 5 COVID-19 ST kits or 5 clinic test referral cards to distribute to contacts in their social network, and testing among their social network contacts was measured. Investigators were masked to study group assignment. Data were analyzed from December 11, 2023, to August 23, 2024.

**Intervention:**

Participants in the intervention group received 5 COVID-19 ST kits; control participants received 5 clinic test referral cards.

**Main Outcomes and Measures:**

The primary outcome was confirmed testing among at least 2 network contacts 8 weeks after randomization. Secondary outcomes included the proportion of participants with at least 1 network contact tested and total number of network contacts reached.

**Results:**

A total of 776 participants (median [IQR] age, 44 [32-57] years; 428 [55.2%] cisgender female) were included in the study, of whom 388 participants were randomized to the ST intervention group and 388 participants were randomized to the control group. There were 112 Hispanic or Latine participants (14.4%), 459 non-Hispanic Black participants (59.1%), and 120 non-Hispanic White participants (15.5%). There was no difference between study groups in the primary outcome, with 5 participants (1.3%) in the ST group vs 2 participants (0.5%) in the control group having at least 2 contacts confirmed tested at the 8-week follow-up (risk difference, 0.0077; 95% CI −0.0056 to 0.0210; *P* = .45).

**Conclusions and Relevance:**

This randomized clinical trial found that secondary distribution of COVID-19 ST kits had no effect on confirmed testing rates among network contacts, which were low in both study groups. Despite these null findings, the study provides insight that may be useful when designing and implementing ST trials.

**Trial Registration:**

ClinicalTrials.gov Identifier: NCT04797858

## Key Points

**Question:**

Can secondary distribution of COVID-19 self-test kits increase testing among medically underserved adults?

**Findings:**

In this randomized clinical trial of 776 adult participants at federally qualified health centers, secondary distribution of COVID-19 self-test kits did not expand test uptake among their social network contacts compared with distribution of clinic test referral cards. In the intervention group, 1.3% of participants had at least 2 network contacts who successfully tested, compared with 0.5% of participants in the control group that received clinic test referrals.

**Meaning:**

This randomized clinical trial found that secondary distribution of COVID-19 self-test kits did not increase confirmed testing among social network contacts.

## Teaser

This randomized clinical trial assesses whether distribution of multiple COVID-19 self-test kits to patients at federally qualified health centers increases COVID-19 testing among social network contacts.

## Introduction

The emergence of the COVID-19 pandemic in 2020 exposed major gaps in equitable access to health care.^1^ Within the US, the pandemic has disproportionately impacted already medically underserved and marginalized communities, with Black and Latine populations experiencing significantly higher rates of infection and mortality compared with White populations.^2,3^ At the same time, in many parts of the US, underserved communities of color have also had lower rates of testing and higher rates of test positivity.^4,5^

Ensuring widespread access to COVID-19 testing has been a key challenge since the beginning of the pandemic. During the initial wave of the COVID-19 pandemic in 2020, access to testing was significantly lower in many parts of the country in underserved communities of color and already socioeconomically vulnerable communities.^2,6^ Despite the emergence of inexpensive rapid antigen tests^7^ and mail-order COVID-19 testing available at no cost through the US government,^8^ testing access has not been equitably distributed.^3,9^ Logistic barriers to obtaining mail-order tests, such as requiring a secure mailing address and needing internet access, may prevent underserved communities from accessing tests.^10^ In many parts of the US, disparities in testing access have persisted, even with the gradual easing of the pandemic and increased availability of testing.^9^

One potential strategy to increase equitable access to testing is the secondary distribution of self-test (ST) kits, by which an individual receives multiple test kits to distribute to contacts in their social networks. This approach decentralizes testing access and can reduce barriers to care, particularly among underserved populations with less contact with health care systems; furthermore, it has been effectively used to expand HIV testing.^11,12,13^ In addition, distribution of testing through known community organizations and health centers can maximize reach in communities with high social vulnerability.^14,15^ To evaluate the effect of secondary distribution of COVID-19 ST kits on testing access, we conducted a randomized clinical trial, the COVID-19 Self-testing Through Rapid Network Distribution (C-STRAND) study.

## Methods

This randomized clinical trial was approved and monitored by the Public Health Management Corporation Institutional Review Board and the University of Pennsylvania Institutional Review Board. All participants provided written informed consent. This study is reported following the Consolidated Standards of Reporting Trials (CONSORT) reporting guideline for randomized clinical trials.

### Study Design and Participants

The C-STRAND study was a 1:1 randomized clinical trial conducted across 4 federally qualified health centers (FQHCs) in Philadelphia, Pennsylvania. Participants, called *index participants*, were randomized to receive either 5 COVID-19 ST kits (intervention) or 5 clinic test referral cards (control) to distribute within their social networks. All FQHCs provided comprehensive primary care to medically underserved populations, including individuals with substance use disorders, complex medical conditions (including HIV and viral hepatitis), individuals with unstable housing, Spanish-speaking populations, and other socioeconomically vulnerable groups. Services are provided regardless of ability to pay, insurance, or immigration status. Recruitment flyers and cards were posted at clinic front desks and waiting rooms. Individuals obtaining on-site COVID-19 testing were also informed of the study. The trial protocol in Supplement 1 has been published in more detail previously.^16^

At the onset of the study, we recruited adult participants who met the following inclusion criteria: age at least 18 years, have a working telephone number, obtaining COVID-19 testing at the time of enrollment, and willing and able to provide informed consent in English or Spanish. Exclusion criteria included individuals who reported prior SARS-CoV-2 infection. Given the high burden of COVID-19 in the community, in August 2021, we modified criteria to no longer limit eligibility to those obtaining COVID-19 testing at the time of enrollment and only excluded individuals who reported a SARS-CoV-2 infection in the past 90 days.

### Interventions and Procedures

Consenting index participants were enrolled and randomized 1:1 to the ST intervention group or the control group using permuted block randomization with varying block sizes stratified by study clinic site. The study biostatistician performed randomization with a computer-generated algorithm, with randomization lists loaded into a data capture system. On randomization, study group allocation was then revealed according to the order of presentation to each study site. In the rare event of computer or internet outage, study staff flipped a coin to determine study group allocation. Investigators were masked, but index participants and study staff were not masked to study group.

After randomization, study staff provided index participants with either ST kits or referral cards to distribute to others in their social network, including (but not limited to) family, friends, and household members, termed *network contacts*. Staff were trained to provide analogous instructions on test distribution and use according to study assignment, without using any motivational interviewing techniques or differential messaging. Staff conducted a baseline survey instrument of index participants, which included sociodemographic characteristics, prior COVID-19 exposure and vaccination, and social network size. Race and ethnicity were self-reported and categorized as Asian or Pacific Islander, not Hispanic or Latine; Black, not Hispanic or Latine; Hispanic or Latine, any race; White, not Hispanic or Latine; and multiracial or other, not Hispanic or Latine (including individuals reporting American Indian or Alaska Native race, >1 race, or other race). Index participants were then contacted 8 weeks following randomization to complete a brief follow-up survey. During the follow-up survey, participants in the ST group were asked how many ST kits they gave out, how many different people were reached, and how many kits they kept for themselves. After the 8-week survey completion, we conducted additional qualitative interviews with 30 randomly selected index participants from each study group to understand barriers and facilitators to distribution and ST kit use.^10^

Index participants assigned to the ST intervention group received 5 COVID-19 ST kits, with kits including a unique referral number linking the kit to the index participant. When the study opened in May 2021 through December 2021, the study used self-collection polymerase chain reaction (PCR) test kits authorized under the US Food and Drug Administration (FDA) Emergency Use Authorization (EmpowerDx), which were sent to a central laboratory with results available in 24 to 48 hours. Self-collection PCR test results were available in a secure web portal, and study staff could then link the network contact to the index participant through the unique referral number. From December 2021 onward, the study used rapid antigen ST kits (Ellume COVID-19 home test, Ellume Health) authorized under the FDA Emergency Use Authorization. The antigen ST kits used a Bluetooth-enabled test developer device, with test results only available through a smartphone app connected to the device.^17^ ST kit packages included a study Quick Response (QR) code linking the ST kit to the index participant. If the network contact clicked the study QR code, test results were then reported to a secure web-based study portal. If network contacts did not click the study QR code, they could still use the ST kit, but test results would not be reported in the study portal. In addition, network contacts could click the study QR code, but decline to participate in the study, and still use the ST kit, with test results not reported in the study portal.

Index participants in the control group received a text message and 5 clinic referral cards that included a study phone number to schedule free clinic-based testing in English and Spanish at study FQHC sites. Referral cards and text messages included a unique referral number linking the referral card to the index participant, which was recorded by study staff if an individual wanted to obtain a clinic-based test. Any individual who called or presented to study sites with a referral card or text message could then be registered for testing. Study staff reviewed all referrals to determine whether the test taker was a unique network contact.

### Outcomes

The primary outcome was the proportion of index participants with at least 2 network contacts who had confirmed testing through the study within 8 weeks following randomization. We chose this outcome because it would represent a sizable expansion of testing access to social network contacts. Secondary outcomes included the proportion of index participants with at least 2 network contacts who tested by the end of the study, the proportion of index participants with at least 1 contact who tested, the number of contacts who tested, and the number of contacts who had positive test results.

We measured testing through 2 objective measures: the primary outcome of confirmed testing and an exploratory outcome of attempted, or initiated, testing. We also obtained self-report measures of test distribution but did not use these data for comparison testing. Testing was confirmed through the study portal for the ST intervention group and through electronic medical records for the control group. For each index participant in the ST group, we defined the number of attempted tests as the total number of clicks (up to 5) on the index participant’s unique QR code. Network contacts who attempted an ST may have completed testing after clicking the QR code, but testing could not be confirmed through the web portal. In the control group, we defined attempting testing as the number of network contacts for a given index participant who scheduled clinic-based testing through the study. At the 8-week follow-up, we asked participants in the intervention group about ST kit distribution behaviors, including the number of ST kits given out, the number of network contacts reached, use of ST kits on self, and unused ST kits still in possession.

### Statistical Analysis

We estimated a sample size of 1048 participants was needed to detect a 10-percentage point difference in the primary outcome with a 2-tailed α = .05 and 90% power. This power calculation was based on 45% in the control group achieving the primary outcome. We made this estimation based on the relatively high societal demand for testing when the study was conceived in 2020. All analyses were performed based on the intention-to-treat principle. Index participants were coded as having zero network contacts testing if no documented tests were registered in the study portal.

The primary analysis used Fisher exact test to compare the proportion of participants in each group that met the primary outcome. We estimated risk differences and 95% CIs between study groups for the primary outcome and secondary outcomes. We used Wilcoxon rank-sum tests to compare counts of attempted and completed testing. All statistical analyses were performed using SAS software version 9.4 (SAS Institute). Data were analyzed from December 11, 2023, to August 23, 2024.

## Results

**Figure 1.**
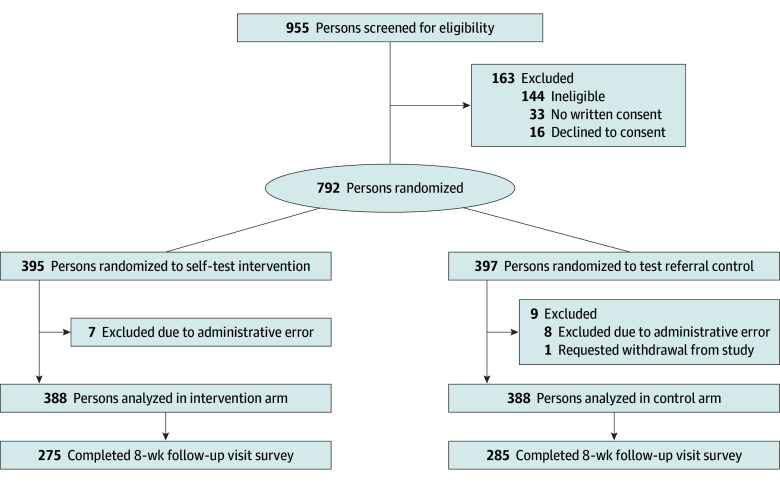
Study Recruitment, Enrollment, and Retention of Participants

Between May 11, 2021, and September 6, 2023, we screened 955 persons for eligibility and enrolled and randomized 792 index participants (Figure 1). We ended recruitment prior to reaching our targeted sample size due to slow enrollment toward the end of the study funding period. A total of 163 persons were not enrolled due to being ineligible, declining to participate, or declining to provide written consent. After randomization, 1 individual requested withdrawal from the study, and an additional 15 participants were withdrawn after enrollment due to a study administrative error with the loss of documentation of signed informed consent. A total of 776 adult individuals (median [IQR] age, 44 [32-57] years; 428 [55.2%] cisgender female) were included in the study analysis, with 388 in the ST intervention group and 388 in the control group (Table 1). Eight weeks after randomization, 275 participants (70.9%) in the ST intervention group and 285 participants (73.5%) in the control group completed the follow-up survey. Their sociodemographic information is reported in eTable 1 in Supplement 1. Follow-up visits were completed by November 15, 2023.

**Table 1.** Baseline Characteristics of Study Participants

Characteristic	Participants, No. (%)		
	Total (N = 776)	Intervention (n = 388)	Control (n = 388)
Age, median (IQR), y	44 (32-57)	44.0 (32-58)	44.0 (32-56)
Race and ethnicity			
Asian or Pacific Islander, not Hispanic or Latine	21 (2.7)	12 (3.1)	9 (2.3)
Black, not Hispanic or Latine	459 (59.1)	221 (57.0)	238 (61.3)
Hispanic or Latine, any race	112 (14.4)	53 (13.7)	59 (15.2)
White, not Hispanic or Latine	120 (15.5)	68 (17.5)	52 (13.4)
Multiracial or other, not Hispanic or Latine^a^	64 (8.2)	34 (8.8)	30 (7.7)
Sex and gender identity			
Cisgender male	328 (42.3)	166 (42.8)	162 (41.8)
Cisgender female	428 (55.2)	212 (54.6)	216 (55.7)
Transgender or nonbinary	20 (2.6)	10 (2.6)	10 (2.6)
Test location			
Site 1	1 (0.1)	1 (0.3)	0
Site 2	429 (55.3)	216 (55.7)	213 (54.9)
Site 3	128 (16.5)	64 (16.5)	64 (16.5)
Site 4	218 (28.1)	107 (27.6)	111 (28.6)
Household members			
Lives alone	184 (23.7)	96 (24.7)	88 (22.7)
1 Other person	167 (21.5)	84 (21.6)	83 (21.4)
2-4 People	252 (32.5)	125 (32.2)	127 (32.7)
5-10 People	61 (7.9)	31 (8.0)	30 (7.7)
>10 People	50 (6.4)	29 (7.5)	21 (5.4)
Unknown	62 (8.0)	23 (5.9)	39 (10.1)
Undomiciled	77 (9.9)	39 (10.1)	38 (9.8)
Education			
<High school	113 (14.6)	50 (12.9)	63 (16.2)
Graduated high school	272 (35.1)	143 (36.9)	129 (33.2)
≥College or more	362 (46.6)	186 (47.9)	176 (45.4)
Unknown	29 (3.7)	9 (2.3)	20 (5.2)
Annual household income, $			
<15 000	201 (25.9)	97 (25.0)	104 (26.8)
15 000 to 49 999	272 (35.1)	139 (35.8)	133 (34.3)
50 000 to 74 999	84 (10.8)	48 (12.4)	36 (9.3)
≥75 000	56 (7.2)	28 (7.2)	28 (7.2)
Unknown	115 (14.8)	58 (14.9)	57 (14.7)
Employment			
Employed	386 (49.7)	199 (51.3)	187 (48.2)
Unemployed	92 (11.9)	55 (14.2)	37 (9.5)
Retired	54 (7.0)	23 (5.9)	31 (8.0)
Disabled	128 (16.5)	54 (13.9)	74 (19.1)
Student	35 (4.5)	20 (5.2)	15 (3.9)
Other or unknown	81 (10.4)	37 (9.5)	44 (11.3)

^a^ Includes individuals reporting American Indian or Alaska Native race, more than 1 race, or other race.

Baseline characteristics of study participants were similar between the study groups. A total of 112 participants (14.4%) were Hispanic or Latine, 459 participants (59.1%) were non-Hispanic Black, and 120 participants (15.5%) were non-Hispanic White (Table 1). Approximately half of all participants were employed (386 participants [49.7%]), more than one-quarter reported household incomes of less than $15 000 (201 participants [27.6%]), 77 participants (9.9%) indicated being undomiciled, and 111 participants (14.3%) reported living with 5 or more other people.

### Primary Outcome

Only 7 index participants met the primary outcome of having 2 network contacts with confirmed testing at the 8-week follow-up: 5 (1.3%) in the ST group vs 2 (0.5%) in the control group (Table 2). There was no statistically significant difference in the primary outcome (risk difference, 0.0077; 95% CI, −0.0056 to 0.0210; *P* = .45). At the end of the study, a total of 10 participants had at least 2 network contacts with confirmed testing, 8 (2.1%) in the ST group vs 2 (0.5%) in the control group (risk difference, 0.0155; 95% CI, −0.0004 to 0.0313; *P* = .11).

**Table 2.** Network Contacts Tested by Study Group

Outcome	Time interval	No. (%)		Risk difference (95% CI)	*P* value
		Intervention (n = 388)	Control (n = 388)		
Primary outcome					
≥2 Network contacts confirmed tested	8 wk	5 (1.3)	2 (0.5)	0.0077 (−0.0056 to 0.0210)	.45
Secondary outcomes					
≥2 Network contacts confirmed tested	End of study	8 (2.1)	2 (0.5)	0.0155 (−0.0004 to 0.0313)	.11^a^
≥1 Network contact confirmed tested	End of study	20 (5.2)	11 (2.8)	0.0232 (−0.0043 to 0.0507)	.14^a^
Exploratory outcome					
≥1 Network contact initiated test	End of study	77 (19.9)	16 (4.1)	0.1572 (0.1129 to 0.2016)	<.001

^a^ For index participant–level outcomes, *P* values were calculated using Fisher exact test between intervention and control groups.

### Secondary and Exploratory Outcomes

When we measured confirmed testing, a total of 20 index participants (5.2%) in the ST group had at least 1 network contact with confirmed testing, compared with 11 index participants (2.8%) in the control group (risk difference, 0.0232; 95% CI −0.0043 to 0.0507; *P* = .14). A total of 47 network contacts had confirmed testing by the end of the study, with 31 individuals in the ST group and 16 in the control group. Three network contacts had positive test results during the study, all in the intervention group (*P* = .24).

Regarding the exploratory outcome of attempted or initiated testing, a total of 77 index participants (19.9%) in the ST group had at least 1 network contact attempt to test, compared with 16 index participants (4.1%) in the control group (risk difference, 0.1572; 95% CI, 0.1129 to 0.2016; *P* < .001). A total of 138 tests were initiated by network contacts by end the of the study, with 116 ST kits initiated and 22 calls made to study sites to obtain a clinic-based test.

**Figure 2.**
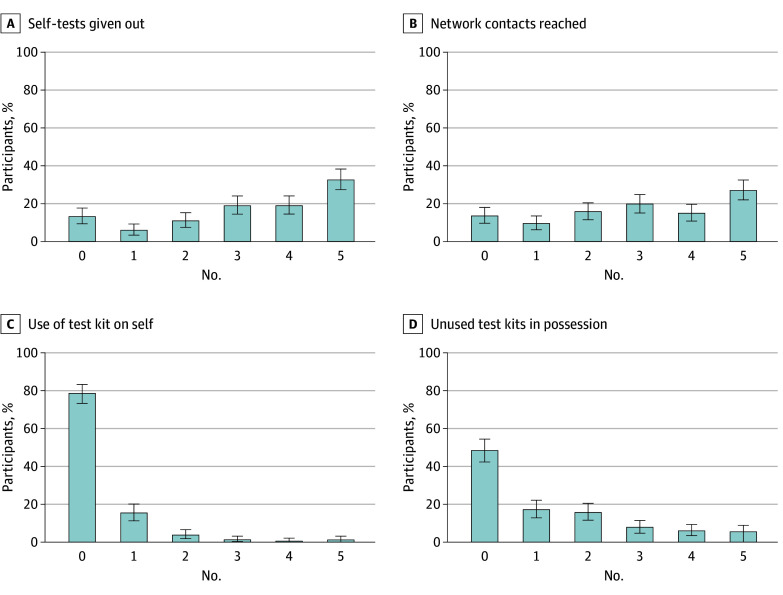
Self-Reported Test Kit Distribution Behaviors at 8-Week Follow-Up in the Intervention Arm (n = 275)

At the 8-week follow-up, 239 of 275 participants (86.9%) in the ST group indicated they had given out at least 1 ST kit since enrollment (Figure 2). Among 239 participants who reported giving out at least 1 ST kit, 223 (81.1%) reported giving the ST kit out to at least 2 different network contacts, a much higher number than suggested by objective measurements. The median (IQR) number of ST kits distributed was 3 (2-4). Approximately 1 in 5 participants (59 participants [21.5%]) reported they had used at least 1 ST kit on themselves in the past 8 weeks. More than half of participants in the ST group (142 of 275 participants [51.6%]) reported still having at least 1 ST kit at the 8-week follow-up (eTable 2 in Supplement 2).

## Discussion

In this randomized clinical trial, secondary distribution of COVID-19 ST kits did not result in increased confirmed COVID-19 testing rates among network contacts. Rates of confirmed testing were significantly lower than expected in both study groups. Although we observed increased test initiation in the ST distribution group compared with the control group, measurement bias may explain these results. While this study did not show an effect of the intervention, this trial can provide valuable insight into practical challenges in measuring ST uptake and studies attempting to evaluate outcomes among social network contacts.

Recognizing the impact of COVID-19 on exacerbating health disparities,^1^ we used a health equity-based approach that focused on enrolling and engaging underserved urban populations at FQHCs, where free or low-cost care is available regardless of insurance or immigration status. In our study, nearly three-quarters of participants were Black or Latine and 1 in 10 participants reported currently being undomiciled. This recruitment strategy may have resulted in lower rates of engagement and follow-up than may have occurred in other populations. Nonetheless, this approach allowed us to recruit populations who have been most impacted by COVID-19, with historically lower participation in clinical trials. In addition, our simple approach in the study intervention is likely widely implementable, given the minimal resources needed to distribute COVID-19 ST kits through health centers.

We hypothesized that our intervention would substantively expand COVID-19 testing access to social network contacts of participants through secondary distribution. Although we observed an association of the intervention with increased network contact testing, there was no effect for our primary outcome. Rates of confirmed testing in both study groups were much lower than initially anticipated, which may have been due to our conservative method of measuring completed testing in both study groups.

Another explanation for the lower-than-expected test uptake was that participants and their social network contacts may have been saving their ST kits for later use. This was supported by our qualitative interviews, which indicated that some individuals may have wanted to save their ST kits for future use.^10^ Another reason for the lower-than-anticipated testing rates may have been fluctuating demand and supply of COVID-19 testing during the pandemic. When we planned the study, rapid antigen testing was not yet widely available, and there were significant concerns about the sensitivity and usability of rapid antigen tests. Within the first year of our study, due to the increasing availability of rapid antigen testing, as well as their widespread adoption, given their practicality and convenience,^7^ we changed the ST kit used in our intervention group to a rapid antigen test. During the second year of the study, COVID-19 testing was more widely available, including free tests made available by the US government in 2022, and it is possible that potential network contacts reached in both groups were less interested in testing through our study.

This study contributes to a small but evolving literature on the importance of focused interventions to reach underserved populations at risk of COVID-19. Despite multiple efforts to expand free and widely available COVID-19 testing, studies have observed persistent disparities in testing among racial and ethnic minority populations and low-income persons. One study in Los Angeles, California, conducted in late 2021 found that low-income persons were less likely to be aware of free testing, and barriers to testing were more prevalent among racial and ethnic minority populations.^9^ Similar to our partnership approach with community-based FQHCs, other studies have highlighted the importance of partnering with community-based organizations in underserved populations to reduce inequities in COVID-19 testing and services.^18,19^

Our research highlights several potential areas for future research. First, additional studies can evaluate whether a secondary distribution approach spurs further engagement with health care systems and services, such as vaccination and primary care. Second, research can evaluate the impact of providing sustained access to ST kits over a longer period. Third, health behavior research can further evaluate the impact of potential messaging strategies to encourage altruistic health behaviors, such as the secondary distribution of COVID-19 ST kits. Finally, future research can focus on identifying objective methods of test uptake that do not rely on self-report yet accurately capture test uptake.

### Limitations

Our study has some limitations. First, we faced substantial challenges in measuring test uptake. We measured test uptake in the intervention group through 2 objective methods and 1 subjective method. All 3 strategies have their limitations. While prior clinical trials of ST kit distribution have relied on self-report to assess test uptake,^20,21,22^ we prioritized the measurement of objective data, either as confirmed test uptake or test initiation. To minimize the potential of social desirability bias, we did not rely on self-report data for comparison testing. Nonetheless, our objective measurements of test uptake had significant limitations as well. While we collected self-report data on ST kit distribution, network contact test uptake, and ST kit use, we were unable to objectively verify whether rapid ST kits had been used by the index participants themselves. Despite our efforts to capture confirmed testing through a QR code–based link, individuals could still use study ST kits without the QR code; therefore, their testing would not be documented by our study. Our measurement of confirmed testing may underestimate the effect of the intervention. We also measured ST initiation through QR code clicks; however, we were unable to confirm whether an individual may have clicked a QR code multiple times, whether it led to a completed test, or whether the index participant had clicked the QR code themselves. The positive effect we observed in our exploratory outcome of test initiation is subject to measurement error and may overestimate the intervention’s impact. Within the control group, network contacts may have been motivated to obtain testing through the referral cards but obtained testing without mentioning the referral card. Because test takers in both study groups could still bypass our objective assessment of test use, it is difficult to ascertain whether the differential assessment of testing by study group would have biased the study results toward or away from the null.

Another limitation was that we only provided 5 ST kits once during the intervention, and participants may have been more likely to distribute ST kits if they had received additional test kits, as our qualitative data suggested.^10^ Furthermore, our study was limited to a single urban setting in the US. Other underserved populations, such as rural residents, speakers of languages other than English, and minoritized populations not well represented in our study may face distinct challenges in access to COVID-19 services. Nevertheless, our study was largely successful in recruiting individuals with high levels of social vulnerability including individuals who were undomiciled, had large household sizes, and/or had low income, as well as Black and Latine individuals.

## Conclusions

Our randomized clinical trial did not show that secondary distribution of COVID-19 ST kits had a substantive effect on expanding testing among social network contacts. Our trial also highlights the substantial challenges in accurately measuring ST uptake, particularly among social network contacts not enrolled in the study. Modifications to our strategy are necessary to augment public health and community efforts to increase COVID-19 test uptake.

## Notes

Supplement 1.
Trial Protocol and Statistical Analysis Plan

Supplement 2. **eTable 1.** Characteristics of Participants at Baseline and 8-Week Follow-Up **eTable 2. **Number of Test Kits or Network Contacts Reached at 8-Week Follow-Up (n = 275)

Supplement 3.
Data Sharing Statement
