# Supplementary material for: Provision of COVID-19 Self-Test Kits to Patients for Distribution to Social Contacts: A Randomized Clinical Trial
Source: JAMA Netw Open. 2025 Jun 4;8(6):e2513708. doi: 10.1001/jamanetworkopen.2025.13708 (PMC12138724; doi:10.1001/jamanetworkopen.2025.13708)
Supplement: Supplement 1. — Trial Protocol and Statistical Analysis Plan [file jamanetwopen-e2513708-s001.pdf]

INDEX

**1** Protocol Background	**6** Participant Payment
**2** Participants	**7** Study Data
**3** Recruitment and Screening	**8** Privacy and Confidentiality
**4** Informed Consent	**9** Risks and Benefits
**5** Study Procedures	**10** References

PROTOCOL OVERVIEW

**Protocol Number**	2004
**Protocol Title**	Secondary distribution of COVID-19 self-tests versus referrals to increase test uptake in underserved populations
**Protocol Version (*mm/dd/yyyy*)**	5/12/2021
**Principal Investigator**	Robert Gross
**PI's Institution/Department**	University of Pennsylvania
**PI's Email Address**	grossr@pennmedicine.upenn.edu
**PI's Phone Number**	215-898-2437
**Funder/Sponsor**	NIH
**Funding Start Date**	11/1/2020
**Funding End Date**	10/31/2022
**Anticipated IRB Closure Date**	

1. PROTOCOL BACKGROUND

1.1 Describe the scientific/scholarly rationale and background for your project. Describe the gaps in scientific knowledge your project is intended to address.

Widespread testing and contact tracing are critical to controlling the COVID-19 epidemic. Like other epidemics, COVID-19 has disproportionately affected marginalized and medically underserved communities. Given growing disparities in the impact of COVID-19, there is an urgent need to increase the reach of testing for SARS-CoV-2, the causative virus and the focus of this study.

The response to the HIV pandemic may be a useful framework to develop strategies to broaden access to tests and follow-up containment efforts. First, based on prior experience with HIV prevention and treatment, it will be advantageous to leverage existing community-partnerships to implement SARS-CoV-2 testing. Second, decentralized testing strategies learned from HIV can be applied to the current COVID-19 epidemic. SARS-CoV-2 self-test kits, approved by the FDA under emergency use authorization, offer an opportunity to expand the reach of testing efforts. Preliminary data show that self-testing is feasible and has excellent sensitivity and specificity.[1] A significant advantage of self-testing is that it reduces the logistical burden and for some, stigma, of venue-based testing.[2,3] Analogously, HIV self-testing has demonstrated remarkable feasibility, acceptability, and efficacy in reaching vulnerable populations across a broad spectrum of contexts.[4-7] However, self-testing may be limited to those with high health care literacy and existing access to health care, without reaching individuals underserved by existing medical systems.

One promising approach to increase test uptake is the secondary distribution of self-tests, where an individual distributes test kits to contacts in their social network and encourages them to self-test. This approach has been

effectively leveraged to increase HIV testing to reach underserved populations who previously had limited prior testing.[4,7,8] By decentralizing testing, this approach does not require individuals to have contact with medical systems, and can lower potential barriers to obtaining testing. An additional benefit of secondary distribution is that it can enhance public health contact tracing efforts,[9] as individuals with COVID-19 can distribute self-testing to close contacts. This approach can increase case detection by facilitating testing among exposed individuals, and potentially ameliorate stigma, fear, and medical mistrust associated with COVID-19 among vulnerable populations because the testing process is decentralized.[10-12]

1.2 List the primary and secondary aims, research questions, and/or hypotheses.

Our central hypothesis is that the secondary distribution of SARS-CoV-2 self-tests can significantly expand test uptake among underserved populations. To test this hypothesis, we will conduct a randomized controlled trial that will assess a self-testing intervention that promotes the secondary distribution of SARS-CoV-2 test kits compared with test referrals. Our aims are:

Aim 1: To determine if the secondary distribution of SARS-CoV-2 self-tests increases test uptake compared with a test referral strategy. Hypothesis: The strategy of distributing self-testing kits will increase test uptake compared with a test referral strategy among social networks of underserved populations.

Aim 2: To determine if secondary distribution of self-tests by individuals with COVID-19 increases case detection compared with a test referral strategy. Hypothesis: Secondary distribution of SARS-CoV-2 self-tests will identify more SARS-CoV-2 infected individuals compared with standard test referrals.

Aim 3: To identify barriers and facilitators to the secondary distribution of SARS-CoV-2 tests. We will use a mixed methods research approach to identify key barriers and facilitators to secondary distribution to inform its future modifications, implementation, and scale-up.

1.3 List the primary and secondary outcomes.

The primary outcome for Aim 1 is test uptake among at least two network contacts per index participant by 8 weeks. Operationally, an index participant is coded as 1 if *at least two of their contacts get tested* and 0 if not, since two additional tests per index will likely lead to substantial growth of testing over time. Secondary outcomes will be: 1) # of unique contacts tested per index by week 8, 2) # of contacts testing positive by week 8, 3) test positivity rate by week 8, 4) proportion of first-time testers, 5) proportion of testers linked to follow-up care, 6) # of self-tests by index participants, 7) total # of tests by network contacts reported at the end of study, and 8) test uptake among at least two network contacts per index participant by end of study.

The primary outcome for Aim 2 is the # of close contacts who test positive for SARs-CoV-2 by week 12. Secondary outcomes include 1) # of contacts tested, 2) proportion of individuals who complete isolation, 3) proportion of individuals linked to follow-up care, and 4) # of new cases identified at end of study.

2. PARTICIPANTS

2.1 Describe the general characteristics of the intended primary participant populations, including age range, gender, sexual orientation, racial/ethnic background, socioeconomic status, health status, criminal history, and any other characteristics relevant to the study.

Study participants will be adults of at least 18 years of age. We will recruit from three Federally Qualified Health Centers (FQHCs) in the PHMC network: The Mary Howard Clinic focuses on providing care to homeless people at all points across the housing continuum. The Ryan White-funded Care Clinic focuses on adults with HIV, viral

hepatitis, and substance use disorders. The Congreso Health Center serves a predominately Spanish-speaking immigrant community in North Philadelphia. Beginning in June 2021, we will also recruit participants from Rising Sun Health Center and PHMC Health Connection, two FQHCs in the PHMC network. Together, the population mix is approximately 47% Black, 35% Latinx, 17% White, and 1% Asian. We anticipate that most individuals will be of lower socioeconomic status and racial/ethnic minorities.

2.2 Select all populations, settings, or records that will be involved in the research. Complete the corresponding appendix for each selection.

☐ People who use substances, substance use treatment sites, and/or substance use records (Appendix J)

☐ Students, school settings, and/or student education records (Appendix L)

For protocols that meet the criteria for exempt research, skip to Section 5.

2.3 Select all intended primary participant populations. Complete the corresponding appendix for each selection.

☐ Pregnant women (Appendix G)

☐ Prisoners (Appendix H)

☐ Children (Appendix I)

☐ People with limited decision-making capacity (Appendix K)

2.4 List the study inclusion criteria.

1) Individuals of at least 18 years of age
2) Able to communicate in English or Spanish and provide informed consent
3) Have a working telephone number

We will no longer require participants to obtain a COVID-19 test to be eligible for the study.

2.5 List the study exclusion criteria.

1) Participation in this trial previously or received COVID-19 testing as part of this study
2) Self-reported prior COVID-19 infection

2.6 Provide justification for the exclusion of broad population groups.

We exclude prior COVID-19 infection due to persistent viral RNA after clinical resolution, and potential misinterpretation of results if individuals re-test in the study to document clearance.

3. RECRUITMENT AND SCREENING

3.1 Identify the expected number of individuals screened and the expected number of individuals consented/enrolled to reach your target sample size. If multi-site, break the total down per study site. If multi-year, break the total down per study year.

We expect to screen an average of 32 individuals per day at the three FQHCs (Congreso, Mary Howard, and Care Clinic) in total. Assuming 25% consent to enroll in our study for an average of 8 individuals per day, it will take a total of 131 days to reach our target enrollment of 1,048 individuals.

3.2 Describe how prospective participants will be identified and recruited. Include information about: how, when, where, and by whom (by position or role, not by name). Describe any recruitment materials that will be used to recruit prospective participants.

Individuals being tested for COVID-19 can be identified and recruited through the call center, through identification in the electronic practice management schedule in the clinics' electronic health system, or through in-person approach of patients when they come in for testing.

Individuals who call the center to schedule COVID-19 testing will be informed of the study. The call center staff will mark the number of individuals screened and the number who are interested in the study. If they indicate interest, their phone number and scheduled testing time will be documented by the call center and given to research staff.

Individuals who are scheduled for COVID-19 testing by the study site will be identified by research staff through the electronic practice management schedule, which provides a list of individuals who are obtaining COVID-19 testing at the study site. This schedule is available in the electronic health system, without accessing individual electronic medical records. The schedule provides individual name, phone number, age, and time and date of testing. No further information will be accessed.

Research staff will review the COVID-19 testing schedule to verify potential eligibility (individual has a listed phone number, and at least 18 years of age), and then use the listed names and phone numbers to call or approach directly in clinic (if they are already in clinic) about the study and assess their interest in participating.

If research staff are unable to contact individuals obtaining testing over the phone, they will send a text message informing the potential participant of the study. If the participant is interested, the research staff can then begin assessing eligibility before obtaining informed consent.

If study sites are able to offer walk-in testing, research staff will screen all potential COVID-19 testers and offer participation to anyone meeting inclusion/exclusion criteria.

In addition to prospectively identifying and recruiting individuals who are seeking COVID-19 testing as described above, we will recruit non-COVID-testing individuals by placing study recruitment flyers in clinic waiting areas inviting interested patients to call or approach study staff to learn more about the study. When permissible at clinic sites, study staff will approach non-testing individuals with information about the study and invite them to participate.

3.3 Describe how prospective participants will be screened. Include information about: how, when, where, and by whom (by position or role, not by name). Describe any instruments that will be used or tests that will be performed during the screening process.

Study interviewers will call those individuals who indicated preliminary interest in being part of the study. They will screen study participants for eligibility and assess if they meet inclusion criteria and if they have any exclusion criteria. They will then provide an overview of the study and initiate the informed consent process over the phone. The interviewers will then conduct the baseline survey over the phone. Finally, the interviewer will provide instructions to the individual to come to the study table after they have received COVID-19 testing.

For individuals who are recruited directly in clinic, study interviewers will assess interest and screen individuals to ensure they meet inclusion criteria.

3.4 Describe the information you will obtain for screening. Explain whether you will retain this as part of the study data.

Study interviewers will indicate all individuals who were assessed for eligibility. We will obtain name and age as part of the screening process.

3.5 Describe how screen failures will be handled and, if applicable, describe the conditions and criteria upon which re-screening is acceptable.

If individuals who are screened are not enrolled in the study, the reason for failure will be indicated by the research staff. These categories include:

1. Did not provide verbal consent
2. Did not meet eligibility criteria
3. Did not complete baseline assessment
4. Did not provide signed informed consent

An individual who has previously declined eligibility can call again and either ask to be part of the study, or re-screened if they call again for COVID-19 testing.

4. INFORMED CONSENT

4.1 Provide a brief summary of the informed consent process by selecting all of the consent procedures you will use and specifying for which study procedures. Complete the corresponding appendix as indicated.

Consent Process	For Which Phase or Activity?
Example: Standard written informed consent	*All study procedures except for focus group activities*
☒ Standard written informed consent	All study procedures except those consented over the phone
☒ Waiver of documentation of consent (Appendix D)	Category 2 network contacts who receive self-test kits consented over the phone; Category 3 network contacts who receive test referrals (Phase 1); Category 4 People who test positive for COVID-19 consented over the phone
☐ Alteration of informed consent (Appendix D)	
☐ Waiver of informed consent (Appendix D)	
☐ Short form (Appendix E)	
☐ Child assent and parental permission (Appendix I)	
☐ Waiver of child assent (Appendix I)	
☐ Waiver of documentation of parental permission (Appendix I)	
☐ Alteration of parental permission (Appendix I)	
☐ Waiver of parental permission (Appendix I)	

4.2 Describe in detail the assent and/or consent procedures in chronological order. Include information about: how, when, where, and by whom (by position or role, not by name).

Consent procedures will differ based on how the individuals are involved in the study. There are four categories of study participants that will be involved in our study: 1) Index participants (individuals randomized to give COVID home test kits or test referrals who enrolled in the clinical trial), 2) Network contacts given COVID-19 self-test kits, 3) Network contacts given COVID-19 test referrals, and 4) Individuals who test positive for COVID-19. The schema is shown below:

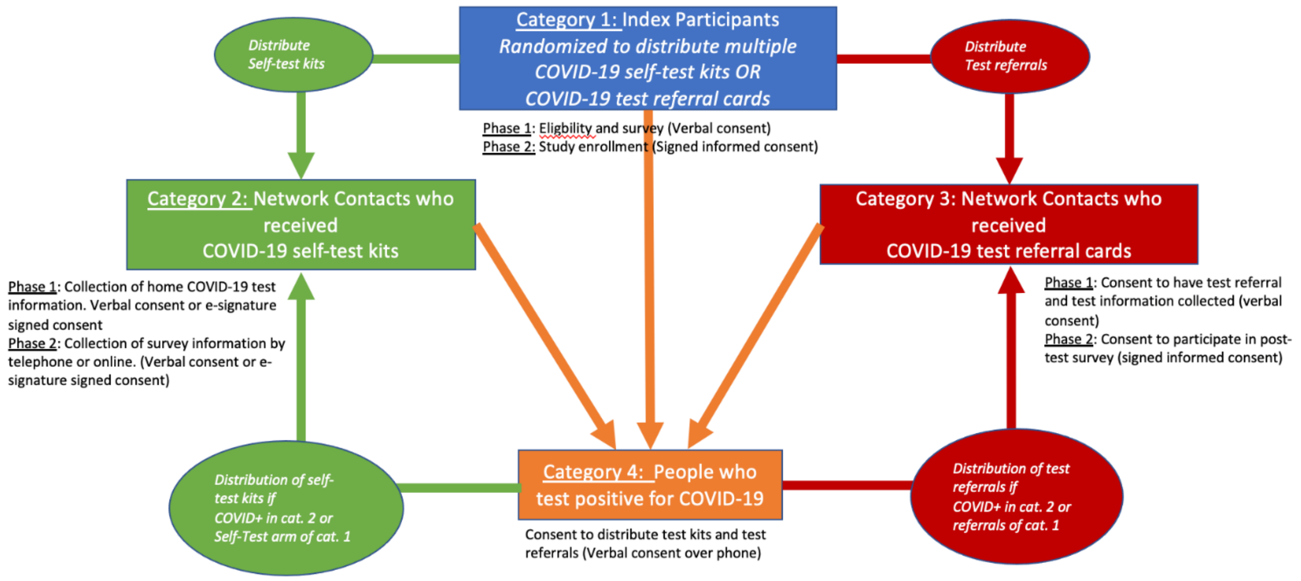

Verbal consent scripts are highlighted in green throughout the protocol and can be found in the Study Scripts appendix.

In addition, this study has been funded as part of the NIH program called the RADx-UP initiative, which stands for Rapid Acceleration in Diagnostics in Underserved Populations. The program stipulates that all studies use common data elements and includes an additional informed consent form to have data shared with the Duke Clinical Research Institute (DCRI), the research group that is tasked with combining data collected from everyone taking part in RADx-UP studies. All consent forms will include additional items at the end to obtain consent for data to be shared with the DCRI.

Category 1: Index Participants – individuals randomized into the RCT.

Description: These are individuals who are recruited and randomized to distribute either self-test kits or test referrals, who are adults 18+, with no prior COVID-19 infection.

Individuals recruited by phone prior to enrollment:

Phase 1.a (phone): Initial consent to participate in study and obtain baseline survey – *verbal consent for individuals recruited over the phone* – see script B "Initial Enrollment – Index Participant")

Individuals who call the call center to schedule COVID-19 testing will be asked if they are interested in participating in the study.

If they are interested, they will be told that a research interviewers will call them. Interviewers will assess eligibility, and then the interviewer will obtain verbal consent to tentatively participate in the study over the phone. The interviewer will then conduct a baseline survey over the phone (see baseline survey document).

They will then be instructed to complete study enrollment when they arrive at the testing sites for Phase 2.

If individuals do not provide full consent in Phase 2, they will not be considered study subjects, and we will only record that an individual was approached and completed the initial survey, but their personal information will not be retained.

Phase 2.a: (In-person) – Completion of enrollment, COVID-19 testing (if applicable), randomization – signed written informed consent

Once prospective study participants arrive at study sites and complete COVID-19 testing (if applicable), study staff will obtain signed informed consent, at which point they will be formally enrolled in the trial, and randomized and assigned to one of two study arms. Individuals will also separately consent to the Duke Clinical Research Institute (DCRI) Study at this time.

NOTE: In order to minimize in-person contact between clinic staff, study staff, and participants, we will obtain survey data from prospective participants after obtaining verbal consent (Phase 1a), but prior to them completing signed written informed consent (Phase 2a). If the prospective participant does not arrive on the date of their scheduled test and study enrollment, we will retain survey data for 30 days after their scheduled test before removing their records from the database.

Individuals recruited in-person in clinics:

Phase 1.b: Initial consent to participate and obtain baseline survey - signed informed consent (in person)

Individuals who are interested will then be assessed for eligibility. If the individual meets eligibility criteria, the interviewer will then provide the brief overview of the study and provide the individual with the informed consent form and be available for additional questions. The individual will then complete the informed consent document for all elements of the consent document, including the DCRI study, at this time.

Once the informed consent form has been signed, the interviewer will provide the individual with a baseline survey.

Covid-19 testing does not have to occur prior to randomization and enrollment.

Randomization and participation in the study will only occur once they have signed informed consent.

Randomization will be stratified by study site and whether individuals received COVID-19 testing at enrollment.

Category 2: Network contacts who received COVID-19 home test kits.

Description: These are individuals who received a COVID-19 home test kit from an Index Participant.

There are two phases of the consent process: Phase 1, consent to have test information collected, and Phase 2, consent to complete a post-test survey. These phases are integrated in the consent process in the electronic consent form.

Phase 1 Consent to have test information recorded – *electronic signature or verbal consent – waiver of documentation requested*

Potential participants must make contact with the study before they submit their sample since test kits cannot be processed by the laboratory unless they are registered. Registration can occur three ways:

(1) online (e-signature)

(2) over the phone (verbal consent)

(3) in-person at study sites (e-signature)

Test kits will all have labels affixed indicated that the tests are part of research, and that by registering the test, test results will be part of a research study. This approach has been used in other studies of HIV home testing and STI testing in the US and internationally.[13-15]

Online registration (e-signature, see RedCap online consent page https://redcap.link/hometest): Test kits will include a link to register the study kit online through the study site. The link will lead the individual through the informed consent process and instruct the individual on how to send-in the test. The individual will then provide an electronic informed consent signature.

Phone registration (verbal consent) – see Script E "Verbal Consent – Network contact (self-test) by phone")*:* For those who register over the phone, research interviewer will document verbal consent for the individual to have test information included.

In-person registration (e-signature, see RedCap online consent page) Individuals can bring home test kits into study sites and have them registered there with the interviewer. The interviewer will provide the individual with a Tablet and the participant will then follow the same online registration process as outlined above. The interviewer will also be available for any assistance needed to register the test. The individual will then provide an electronic informed consent signature.

Phase 2: Consent to participate in survey – *electronic signature online or verbal consent over the phone.* $15 study compensation.
During the Phase 1 consent process, individuals will be asked if they are interested in completing an optional survey. Individuals will be notified that they can decline to participate in the survey and still obtain testing through the study. If individuals consent to participate in the survey, they will be given the option of completing the survey after test registration. The survey will be online but will also be conducted over the phone by interviewers if preferred by participants. Upon completion of the survey, the participant will indicate how they would like to receive their $15 study compensation, either as a gift card picked up at a study site or a virtual payment. They will also be offered the DCRI study at this time.

Category 3: Network contacts given COVID-19 test referrals.
Description: These are individuals who received a COVID-19 test referral number and/or a referral card from an Index Participant.

Phase 1 Consent to have test referral and test information collected – *verbal consent over the phone* – see script F "Verbal Consent – Network contact (test referral) by phone"

Network Contacts who receive test referrals will be given instructions on how to obtain COVID-19 testing through PHMC through referral cards and/or text messages. These referral cards will have a referral number and they will be instructed to give this number when scheduling the test. When they call the study number to schedule a test, research interviewers will ask for the referral number. The research interviewer will ask for verbal consent to include their test referral in the research study.

If the Network Contact declines consent, the interviewer will only mark that a phone call was made to schedule a test but will not retain the test referral number or any PHI. If the Network Contact provides verbal consent to have the number and PHI retained, the interviewer will record the patient's scheduled test in the research database. Only the patient's medical record number and test referral number will be included in research database for test tracking purposes. The research interviewer will then track the test referral to see if the Network Contact completed testing.

After test registration, the research interviewer will offer to conduct an optional survey over the phone if the Network Contact has provided verbal informed consent. The survey can be completed at any time within one week after testing. Once the survey is completed, the Network Contact can obtain the $15 compensation at the time of testing.

NOTE: As with Index Participants, in order to minimize in-person contact between clinic staff, study staff, and participants, we will obtain survey data from prospective participants after obtaining verbal consent, but prior to them completing signed written informed consent. If the prospective participant does not arrive on the date of

their scheduled test and study enrollment, we will retain survey data for 30 days after their scheduled test before removing their records from the database.

Phase 2 Testing and signed informed consent.
Network Contacts who present for testing will then complete signed informed consent. Individuals will also be offered the DCRI study participation and consent form at this time. If they have already completed the survey, they will be offered the $15 compensation gift card at that time. If they have not completed the survey but consented to do so, they will be offered the survey then or within the next week, and can pick up the card or receive virtual payment after completion.

Category 4: COVID-19 positive participants (Network Contacts)
Description: All study participants in Categories 1-3 who test positive for COVID-19 will be eligible to be part of a COVID-19+ cohort, who will be asked to distribute test kits or test referrals to their close contacts. Index participants who test positive will not need to re-consent to be part of this cohort. Network Contacts in categories 2 (self-test arm) and 3 (test referral) arm who test positive will be asked to provide additional informed consent to safely distribute test kits or test referrals. Individuals will be assigned test kits or test referrals based on which arm of the study they were enrolled in the study (e.g., if Network Contacts received a self-test and tested positive, they will receive additional self-test kits, if they received a test referral and tested positive, they will receive additional test referrals.).

Consent to distribute self-test kits – *verbal consent over the phone* – see script J "Informed Consent for COVID-19+ network contacts participant, Self-Test arm"

Consent to distribute test referrals – *verbal consent over the phone* – see script K "Informed Consent for COVID-19+ network contacts participant, Test Referral arm"

Interviewers will call all individuals who test positive and provide guidance on symptom management, isolation, close contacts. and invite Network Contacts who tested positive to participate in the study by safely distributing additional test kits or test referrals.

If individuals do not provide consent to participate in this cohort, they will still receive standard post-test counseling.

4.3 Describe any measures to ensure or test participants' understanding of the information presented during the informed consent process.

As previously described, Index Participants (Category 1) will undergo two phases of informed consent with research staff. Research staff will be available to answer questions throughout the process. A hotline will be available for answering general study questions.

Network Contacts reached through the test referral arm (Category 2) will also undergo two phases of informed consent with research staff. Research staff will be available to answer questions throughout the process and ask open-ended questions to ensure participant understanding of the information presented.

Network Contacts reached through the self-test arm (Category 2) will have several options to enroll in the study. An electronic informed consent form will be available to them in both English and Spanish. Online prompts will be posted to ask participant understanding in addition to the electronic signature. Network Contacts who register for self-test kits over the phone will undergo a verbal consent process, and research staff will be

available to answer questions throughout the process and ask open-ended questions to ensure participant understanding of the information presented. A study hotline will also be available for any questions.

For all other Phases and procedures, research interviewers will make the determination that the individual has full capacity to provide informed consent during the enrollment process. The interviewer at Congreso Health Center will be bilingual in English and Spanish to ensure Spanish-preferring individuals understand the information presented. A study hotline will be available to all participants answer any questions.

4.4 If a child who provided assent to participate in the study becomes 18 years old during the course of the study, describe the process to re-consent the participant.

All subjects must be 18 years or older, so children will not be involved in this research.

1. STUDY PROCEDURES

5.1 Provide a detailed description of the study procedures in chronological order. As applicable, include information on: differences between the control and experimental groups, the number of follow-up visits, study visit windows, participant time commitment per activity, and the setting/location per activity.

1. *Recruitment, Eligibility assessment, and Enrollment of Index Participants:*

We will recruit over the phone and in-person.

Phone recruitment:

Individuals who call the call center to schedule COVID-19 testing and vaccination will be asked if they are interested in participating in the study.

If the individual states they are not interested, the call center personnel will mark that an individual was approached but declined participation at that stage.

If the individuals states they are interested, the call center personnel will mark the individuals' personal information for the study researcher to call them to assess eligibility.

Eligibility assessment

The research interviewer will then call the individual who indicated interest. If they are interested, interviewers will assess eligibility through the following questions:

1. "Are you 18 or over?"
2. "Do you have a working telephone number?"
3. "Have you ever tested positive for COVID-19 in the past?"
4. "Have you received a test as part of a COVID study at PHMC?"

If the individual answers "No" to Items 1-2, or "Yes" to Items 3-4, the RA should mark "*ineligible*" and indicate the reason for ineligibility

If the individual meets inclusion criteria, the interviewer will obtain verbal consent to tentatively participate in the study over the phone.

Enrollment (Week 0)

Part 1: The interviewer will then conduct a baseline survey over the phone (see baseline survey document). The survey is anticipated to take approximately 15-20 minutes. If the participant is interested but cannot complete the survey at the time, the interviewer will call back at an agreed upon later time. Upon completion of the

survey, the interviewer will then instruct the potential participant to come to the study table after completing testing for completion of enrollment.

For patients that are recruited on-site, the interviewer will obtain <u>written informed consent at this stage.</u> After completing informed consent, the study subject will then complete a baseline survey on a computer tablet to minimize direct in-person interactions.

<u>Phase 2:</u>

Once prospective study participants arrive at study sites, study interviewer will complete consent process and obtain signed informed consent, if they have not provided already (e.g., participants that were recruited on-site and have already provided informed consent).

Once informed consent has been completed, individuals will be formally enrolled in the trial, and randomized and assigned to one of two study arms.

The interviewer will complete the randomization and provide the Participant, deemed *Index Participant*, either 5 home test kits, or test referral cards and a text message with test information and the test referral number.

<u>Self-test kit arm procedures:</u>

If randomized the self-test kit arm, the interviewer will retrieve a <u>package of 5 self-test kits</u>.

Each package will be labeled with a 5-digit *package* number beginning with 1 (to indicate the self-testing arm) <u>separate</u> from the subject's ID number. These 5 digits will be test package ID that will be linked to the subject ID.

Each self-test kit in the package will then be consecutively labeled 1, 2, 3 and so on.

The interviewer will then enter this 5-digit package number into that subject ID's record.

Self-test kits will be kept in secure locations determined by the staff of the participating clinic sites.

Self-testing kits:

Each self-test kit in the package is consecutively labeled 10001-1, 10001-2, 10001-4, and 10001-5.

The interviewer will provide an explanation of how to distribute test kits to contacts.

Intervention instructions

Participants will be encouraged to distribute the self-tests to individuals in their social network, including family members, with encouragement to deliver to network contacts age 18 and over with the following:

1) A known exposure to COVID-19. If exposed, testing should ideally be done 5-7 days after the exposure.

2) Are symptomatic (fever, difficulty breathing, new cough, unusual fatigue, loss of taste or smell, rash on fingers or toes)

3) Are believed to be at high risk of COVID-19 due to multiple in-person interactions with others.

The Index Participant will also be instructed on how to register test kits, which can be done either online, over the phone, or in-person.

<u>Test referral Arm procedures:</u>

If randomized to the test referral cards, the interviewer will retrieve a package of 5 test referral cards with guidance on distributing test referrals. They will also receive a text message with simple instructions to send to contacts on how to test, which they can then copy and send to contacts (See Script N.1. "Referral for Index Participant in Test Referral arm."

Each package will be labeled with a 5-digit package number beginning with 2 (2 indicates the control arm) separate from the subject's ID number. Each test referral card in the package will then be consecutively labeled 1, 2, 3 and so on.

The interviewer will then enter the 5-digit package number into that patient's record.

Test referral cards will be kept in secure location at clinic sites.

Test referral cards:

- Each test referral card the package is consecutively labeled, e.g. 20001-1, 20001-2, 20001-4, and 20001-5.
- Test referral cards will have *bilingual* clinic information on where to get testing and instructions to return the card to the interviewer in each of the clinic sites. If the interviewer is unavailable at the time, the subject can also return the card to the front desk staff.

Instructions:
Participants will be encouraged to distribute COVID-19 testing referral cards and forward the text message referral to individuals age 18 and over in their social networks, including family members, with encouragement to deliver to network contacts with:
1) a known exposure to COVID-19. If exposed, testing should ideally be done 5-7 days after the exposure.
2) are symptomatic (fever, difficulty breathing, new cough, unusual fatigue, loss of taste or smell, rash on fingers or toes)
3) believed to be at high risk of COVID-19 due to in-person interactions with others.

Participants will be recommended to wear fitted masks while interacting with others and wash hands during in-person contact with others. Participants will be encouraged to minimize contact with others during distribution, and to limit in-person contact whenever possible. Potential distribution strategies include "contactless pickup" such as leaving outside rooms, buildings, in mailboxes, or outdoor distribution. Participants in the test referral arm will also be given the option to send a text message referral, which can be done remotely.

Index Participants
- will be contacted again by study interviewers if their test returns positive.
- Will be contacted again in 8 weeks to complete a follow-up online survey, lasting approximately 15 minutes. If this is not completed by week 10, a study interviewer will call them to conduct the survey over the phone. If they complete it, they will get an additional $15 loaded to their gift card.
- May be contacted to participate in a telephone interview.

Finally, the interviewer will provide them with a $15 gift card and instruct them to hold on to it after use for later recharge and reuse.

2. *Follow up of index participants*

At week 8, index participants will receive a text message from study staff (See Script N.2 "Text message for Index Participants at 8-week follow-up") indicating that it is has been 8 weeks since they registered for the study. The text message will include a link to an online survey. See 8-week follow-up survey.
If the survey is completed online, interviewers will be notified and will add an additional $15 to the gift card of the Index Participant. If they have lost it, they can pick up another one at one of the study sites.

A reminder text message will be sent 3 days later and 7 days later if they have not completed the survey.

If the index participant still has not completed the survey at week 9, the interviewer will call them to complete the survey. The interviewer will call two more times at weeks 10 and 11 if they are still unable to reach the Index Participant.

Network Contacts

3. *Network Contacts in the self-testing arm:*

If self-test kits are used, testing results will be transmitted back to PHMC for clinical follow-up with the linked test kit ID.

<u>Online registration</u>:

Individuals can register the test kit online through our study website, which will guide them through the informed consent process and provide information on how to conduct the self-test and return the self-test. The testing partner will transmit results directly to the patient and PHMC once they become available.

<u>Phone registration</u>:

Individuals can also register test kits over the phone with study interviewers to become Network Contacts. The interviewer will complete the informed consent process (as above) and assist the individual with registering the test.

The Network Contact can then drop off the test at study sites to be mailed in or mail the test in directly.

<u>In-person registration</u>:

Individuals can also register test kits in person at study sites by bringing test kits to study sites.

Interviewers will register individuals as Network Contacts. Interviewers will supervise self-collection but cannot perform the test themselves.

The interviewer will then complete the informed consent process.

The interviewer will then mail the test kits in.

4. *Network Contacts in the test referral arm*:

Individuals who receive referral cards or texts will have a referral number and they will be instructed to give this number when scheduling the test. When individuals call the study number to schedule a test, research interviewers will ask for the referral number. The research interviewer will ask for verbal consent to include their test referral in the research study.

If the individual declines consent, the interviewer will only mark that a phone call was made to schedule a test but will not retain the test referral number or any PHI.

If the individual provides verbal consent to have the number and PHI retained, the interviewer will record the individual's scheduled test and their name into the database to be tracked.

Over the phone, the research interviewer will also indicate that when Network Contacts present for testing, they can complete a post-test survey on-site after testing and receive a $15 study compensation and should arrive at the study table after they obtain on-site testing.

Network Contacts who present for testing will be offered a post-test survey after completing testing. Because interviewers will have a schedule of when Network Contacts have scheduled testing, interviewers will also approach Network Contacts when they arrive for their scheduled testing.

If a Network Contact approaches the study table at the testing site, the interviewer will look up their name to document that they completed on-site testing, complete the informed consent process, and then provide Network Contacts with a Tablet computer to complete a post-test survey.

After completion of the post-test survey, the interviewer will give the Network Contact a $15 gift card. The interviewer will remind the Network Contact that they will reach back out for participation in future surveys.

Daily review of test results

Research interviewers will be responsible for reviewing all self-test results and clinic test results on a daily basis.

Review of self-test results

Self-test results:

1. Positive test results will trigger a phone call (see Script I "Communication of test results – COVID-19 positive" for COVID-19 positive individuals.
2. Negative test results will trigger a phone call (see Script H "Communication of test results – COVID-19 negative")
   a. If the individual has already completed a post-test survey, they will receive a phone call from PHMC researchers indicating their negative test result, and provide guidance on test interpretation and offer further linkage to clinical care if needed.
   b. If the Network Contact *has* not completed a post-test survey: the interviewer will call the Network Contact, inform them of their test result, and also offer to conduct the post-test survey.

Review of clinic test results:

1. The interviewer will be responsible for contacting all Network Contacts with test results. At the beginning of each work day, they will review all new test results of Network Contacts who obtained testing through PHMC.

Test Results:

2. Positive test results – will follow the script as indicated below for COVID-19 positive individuals.
3. Negative test results – Interviewers will call the Network Contacts and inform them of negative test results, provide guidance on test interpretation, and offer further linkage to clinical care if needed.

Management of positive COVID-19 test results:

Summary:

All individuals who test positive for COVID-19 during the trial require contact for follow-up. Here, we lay out procedures depending on *who* is testing positive.

COVID-19 participants are divided into two groups: ST participants (those in the Self-Testing arm) and TR participants (those in the Test Referral arm).

<u>*COVID-19 participants Protocol*</u> (See Script I "Communication of test results – COVID-19 positive")

- If someone else picks up, ask for best way to get ahold of contact or best time to call back.
- Make at least one attempt within 24 hours of test result receipt.
- Each attempt = 2 back-to-back calls with a voicemail left.
- If no voicemail is available, try at least 2 additional times within 48 hours of test result receipt to reach them.
- Interviewers should make at least 3 attempts within 72 hours of assignment
- At least 4 attempts in one week
- If the individual is unable to be reached, you may mark "Unable to reach" as the final disposition.
- NEVER reveal test results to anyone else without first confirming identity.

RAs will call all individuals who have positive test results and follow the script below. There are five goals of the call:

1. Assess COVID-19 symptoms
2. Escalation of care and referral to PHMC
3. Isolation guidelines
4. Contact elicitation – procedures outlined as described below
5. Offer additional test kits or referrals as part of the research study.

RAs will ask if the Network Contact would like to participate in an ongoing study of COVID-19 testing that would provide them with additional self-test kits for close contacts.

- If the Network Contact provides informed consent, the Network Contact then becomes a "COVID-19 ST Participant" (see Scripts J "Informed Consent for COVID-19+ network contact participant Self-Test arm" and Script K "Informed Consent for COVID-19+ network contact participant test referral arm")
- Individuals will be informed that by contacting their close contacts and asking them to get tested, they may be asked to tell them about their COVID-19 infection.

If the COVID-19 participant is in the Self-testing arm (COVID-19 ST): (see Script L "Contact elicitation for COVID-19+ participants ST arm")

- The interviewer will ask individuals about close contacts and will offer up to 3 additional self-test kits to distribute to close contacts.
- The interviewer will elicit close contact information, and help the individual prioritize who to give testing to.
- The interviewer will instruct COVID-19 participant to tell close contacts to quarantine, monitor symptoms, and that if desired, they can obtain testing at PHMC clinics and receive a $15 card if they do so.
- The interviewer will then send the COVID-19 participant a "COVID-19 referral text" (See Script N.3 "Text message for COVID-19 positive contact self-test kits") that they can forward to contacts.
- The interviewer will then conduct the COVID-19 Participant survey over the phone.

Delivery of self-testing kits to close contacts:

- Self-test kits can be obtained through the following strategies:
  1. Shipped directly to the COVID-19+ subject – recommended if close contact is a *household member*
  2. Arranged for pick-up at one of the 3 PHMC clinic sites. – Registration can be completed over the phone (Script E: "Verbal Consent – Network contact ST phone")

If the COVID-19 participant is in the test referral arm: (see Script M "Contact elicitation for COVID-19+ participants TR arm")

- The interviewer will elicit close contact information, and help the individual prioritize who to give testing to.
- The interviewer will instruct COVID-19 participant to tell close contacts to quarantine, monitor symptoms, and that if desired, they can obtain testing at PHMC clinics and receive a $15 card if they do so.
- The interviewer will then send the COVID-19 participant a "COVID-19 referral text" (See Script N.3 "Text message for COVID-19 positive contact test referral") that they can forward to contacts. There is no limit to the number of text messages they can send.

Follow-up of COVID-19 participants

COVID-19 participants will be sent an additional survey at 8 weeks to assess their experience with COVID-19, isolation, and care follow-up.

Study in-depth interviews

After the end of their follow-up period, Index participants and network contacts will be randomly selected to participate in in-depth interviews. Interviews will be semi-structured and conducted over the phone by study interviewers. Open-ended questions will ask about experiences distributing and receiving COVID-19 test kits and

test referrals, changes in life due to COVID-19, and social networks. Interviewers will take notes through the interviews, but these will not be audio recorded.

<u>Translation of study materials into Spanish (Informed Consent forms, study measures, and recruitment materials)</u>

All informed consent forms, study measures, intervention materials, and recruitment materials will be translated into Spanish (Latin American) professionally through ALTA Language Services, or another professional translation service, if ALTA is unavailable to perform the translation. ALTA language services provide a notarized certification of translation completion and accuracy upon completion of translation services.

Common data elements from the Duke Clinical Research Institute have already been translated by that national research group into Spanish.

For day-to-day operations and materials that may need to be translated or interpreted on an ad hoc basis, these will be performed by our bilingual interviewer. If feasible, the translation will be checked for veracity, proofing, and editing with another bilingual individual.

5.2 Briefly describe the sources or measures that will be used to collect data about participants. As applicable, include information on: instruments or surveys that will be administered, tests that will be performed, and the time points when each data will be accessed or obtained.

Data collected directly from participants:

Baseline data from study participants, including both index participants and network contacts will be collected upon enrollment. These data include required common data elements (items required by the NIH parent grant).

An endline survey will be conducted 8 weeks after enrollment among Index Participants. Follow-up measures will assess who individuals distributed testing to and experiences distributing testing.

Among individuals who test positive for COVID-19, an additional survey will be conducted at 8 weeks.

Data collected from other sources (identify each source; e.g., health care provider, parent, public records):
Health care records from index participants: COVID-19 test results, medical visits from PHMC electronic medical record.
Health care records from network contacts who received test referrals: COVID-19 test results, medical visits from PHMC electronic medical record.
Health care records from network contacts who received COVID-19 home test kits: COVID-19 test results from test partner, and medical visits from PHMC electronic medical record.

5.3 Describe pre-defined criteria for removing participants from the study and the procedures for informing participants of their removal from the study. Include information on the handling of their data and measures to ensure individuals' safety and privacy after ceasing research activities.

There are no protocol defined criteria for removing participants from the study. If study participants indicate that they wish to cease participation, they can do so at any time after enrollment by informing any of the research staff. The staff will then ask the participant if the data they have already contributed can continue to be used with the same protections in place. If yes, we will include their data in the analysis since this is a strategy trial. If they wish to be entirely removed from the study, the staff will search the database for any participant

information and remove their data from the electronic database. All paper copies of the informed consent forms will be shredded.

For protocols that meet the criteria for exempt research, skip to Section 7.

5. PARTICIPANT PAYMENT

6.1 Describe any <u>compensation</u> that will be given to participants for the completion of research-related activities. Include the monetary value and type of compensation per activity.

Index Participants will be given a debit card with $15 loaded onto the card upon completion of the baseline survey and COVID-19 testing. An additional $15 will be added to the card if they complete the end-line survey at 8 weeks. Value can be added remotely to the debit cards.

Network Contacts will be given a debit card with $15 loaded onto the card upon completion of the survey. An electronic virtual payment option will also be available if preferred.

Individuals who test positive for COVID-19 will be approached for participation in the cohort study of self-test kits vs. test referrals. They will be given a debit card with $15 loaded onto the card if they agree to participate.

6.2 Describe any <u>reimbursements</u> that will be given to participants to repay them for costs they may have incurred over the course of the study. Include the monetary value and type of compensation per activity.

No reimbursements will be given to participants.

6. STUDY DATA

7.1 Select all of the personal identifiers you will access and obtain during this study.

Access *means to view or to perceive data, but not to possess or record it.* ***Obtain*** *means to possess or record in any fashion (writing, electronic, video, email, voice recording, etc.) and to retain for any length of time.*

☒ Names
☒ Geographic subdivisions smaller than a State (street address, city, county, precinct, ZIP code, etc.)
☒ Dates related to an individual (date of birth/death, date of admission/discharge, date of service, etc.)
☒ Telephone numbers
☐ Fax numbers
☒ Electronic mail addresses
☐ Social security numbers
☒ Medical record numbers
☐ Health plan beneficiary numbers
☐ Account numbers
☐ Certificate/license numbers
☐ Vehicle identifiers and serial numbers
☒ Device identifiers and serial numbers (test kit numbers and referral card numbers)
☐ Web URLs
☐ Internet protocol (IP) addresses
☒ Biometric identifiers including fingerprints and voiceprints (e.g., audio recordings)
☐ Full face or comparable photographs/images
☐ Any other unique identifying number, characteristic, or code

7.2 Does your study involve PHI and need to comply with HIPAA regulations?

HIPAA regulations apply to covered entities that access, collect, use, or disclose protected health information (PHI). PHMC is a HIPAA-covered entity and thus, ***any study that involves PHI must comply with HIPAA regulations****. HIPAA only covers <u>identifiable health information</u>. Studies that involve only (A) health information*

without any of the 18 personal identifiers listed above or (b) non-health related information that contain identifiers do not need to comply with HIPAA because neither form of data meets the definition of PHI. Use the guide below to verify if your study involves PHI.

All or some study data:

(a) Contain one or more of the 18 personal identifiers.	☒ Yes ☐ No	If you answered "No" to (a), then HIPAA regulations do NOT apply.<br><br>If you answered "Yes" to (a) **AND** (b), (c), **or** (d), then **HIPAA regulations apply to this project.** Submit Appendix C.
(b) Relate to an individual's past, present, or future physical or mental health or condition.	☒ Yes ☐ No	
(c) Relate to an individual's past, present, or future provision of healthcare.	☒ Yes ☐ No	
(d) Relate to an individual's past, present, or future payment for the provision of healthcare.	☒ Yes ☐ No	

7.3 Is there a possibility that *de-identified* information or biospecimens will be used for future research studies or distributed to another investigator for future research studies without additional informed consent from participants?

This only applies to studies that prospectively collect information or biospecimens. Select "No" if your study involves secondary analysis only.

☒ Yes, de-identified information or biospecimens may be used or distributed for future research.

☐ No, de-identified information or biospecimens will not be used or distributed for future research.

7. PRIVACY AND CONFIDENTIALITY

8.1 Describe procedures to protect participants' privacy *during* the study (i.e., precautions you have taken to protect the participant from being recognized as a research subject).

Study participant's privacy may be threatened during the study when they are engaging in research activities. One of the significant potential strengths of the intervention we are studying, testing at home, is increased privacy. For participants who receive the self-test kit intervention, a significant benefit of this intervention is the increased privacy of test-taking. Although this has not been studied for COVID-19 self-testing, prior research in self-testing for HIV, syphilis, chlamydia have demonstrated that individuals feel privacy, anonymity, and confidentiality are enhanced with self-testing compared with standard of care facility-based testing.

To minimize logistical barriers to participation, we have included multiple ways individuals can have tests and surveys conducted, including in person, over the phone, and/or online. In-person contact with research team will be limited at study sites as much as possible, and we will utilize strategies such as electronic tablet surveys, electronic signatures, in order to minimize direct contact with study staff. We have included multiple procedures in our protocol to protect participants' privacy during the study tailored to each contact method.

For in-person research activities at study sites, research tables will be set apart from the clinic in private areas of the clinic (e.g., private rooms), or if testing is conducted outside as at the Congreso Health Center, in a separate area) where participants' privacy can be maximized. Only one study participant will be allowed to directly interact with study staff at a time. Participants can complete study questionnaires via electronic tablet to maximize privacy.

For contact made over the telephone, research interviewers will initially identify themselves only as calling from PHMC and will not describe any research activities over the phone, until after individuals have identified themselves over the phone. Interviewers will then ask the participants if they are in a private location where they feel comfortable to discuss study materials.

Online interactions will be initiated by the participants themselves, which means they will control their own privacy during the study. If they are not able to maintain privacy this way, they also have the options of phone and in-person as above.

8.2 Describe all of the procedures you will use to protect participants' confidentiality *during* the study (i.e., precautions you have taken to protect participants' data from being unnecessarily and inappropriately disclosed; e.g., authorization of access, password protection, encryption, physical controls, certificates of confidentiality, separation of identifiers and data). Include information on any measures that will be taken during storage, access, use, and transmission of study data.

If you will obtain information on participants' substance use disorder from a treatment provider, then only answer this question for all other data not related to substance use records (e.g., self-report data). All confidentiality and data security questions around substance use records should be recorded in Appendix J.

Since self-report and medical data will be collected and stored as part of this study, it is possible that study participants' confidentiality can be threatened during the study. Only PHMC research staff will be authorized to access to participants' contact information data. PHMC research staff are routinely trained in research ethics and the maintenance of confidentiality. This training will be reinforced prior to initiation of the trial. Additionally, the PENN Clinical Research Computing Unit (CRCU) Data Management System has set up several safeguards to prevent unauthorized access to study data by non-PHMC staff or others. An automatically generated index number is assigned to a subject's study identification number (unique for each patient). A linked subject identification table is created for the storing of subject name, address and telephone contact information. This table uses the automatically generated index number rather than the study identification number. The master subject map and subject identification information tables are maintained in a separate database. Only the PHMC staff will have access to this table for purposes of linking to contact information to clinical data in order to communicate results of COVID tests to participants. Using this method, the PENN investigators cannot link contact information to medical information or other study data. We have long-established protocols to guard against improper use of hard copies of data (e.g., locked files, numeric coding procedures). The present research team has not experienced the unauthorized use of study data. A server-based data collection procedure with state of the art encryption and authentication procedures based on decades of experience by the CRCU managing clinical research project data will minimize the possibility of loss of privacy or confidentiality.

All quantitative data will be numerically coded and information linking the numeric code to the subject's name will be kept in a secured file cabinet and office. In addition, computer data files will be stored on password-protected computers and communication among the staff will use participant code numbers, not names. No information concerning data will be presented with participant names.

8.3 If identifiers will be obtained, indicate how the identifiers will be stored.

If you will obtain information on participants' substance use disorder from a treatment provider, then only answer this question for all other data not related to substance use records (e.g., self-report data). All confidentiality and data security questions around substance use records should be recorded in Appendix J.

☐ Not applicable; identifiers will not be obtained.

☒ Identifiers will be stored separately from study data and a key or code will be kept.

Describe where the data study, identifiers, and key will be stored, and state the retention period:

Study data, identifiers, and keys will be stored and retained for 7 years after completion of the study (lock of the dataset). Data will be securely stored by the UPenn CRCU, which has extensive experience managing data for clinical trials. Consent and HIPAA documents will be stored securely on PMACS servers managed by the Penn CRCU.

☐ Identifiers will be removed or destroyed.

Describe how identifiable data will be de-identified or destroyed. Include information on how, when, and by whom (by role or position, not by name):

☐ Identifiers will not be removed or destroyed.

Provide the rationale for retaining identifiable data and state the retention period. Include information on how and where the identifiable information will be stored:

8. RISKS AND BENEFITS

9.1 Select all of the <u>reasonably foreseeable</u> risks of harm, discomforts, and hazards to the participants and others as a result of study participation.

☒ **Breach of confidentiality** to records containing identifiable private information or biospecimens
☐ Disclosure of participants' responses outside of the research context could place the subjects at risk of **criminal or civil liability**
☐ Disclosure of the participants' responses outside of the research context could be **damaging** to the participants' financial standing, employability, insurability, education, reputation, social relationships, services, or eligibility services
☒ **Psychological discomfort or distress** from providing or being exposed to personal or sensitive information
☒ Possibility of or perceived **invasion of privacy** to the participant or their family
☐ Possibility of or perceived **coercion or undue influence** to participate in the study
☐ Presentation of materials which some participants may consider **sensitive, offensive, or threatening**
☐ **Physical or psychological harm** such as pain, injury, or disease including side effects from drugs and devices
☐ Other (please specify):
☐ No risks

9.2 For each risk selected above, describe the magnitude, probability, duration, and/or reversibility of the harm, discomfort, or hazard.

<u>Breach of confidentiality</u>: While a breach of confidentiality to records containing identifiable information is possible, the probability is low given the numerous safeguards we have taken to protect participant data and identifying information. The present research team has not experienced the unauthorized access of study data. Although our study will involve sending biospecimens to third party laboratories for analysis, no biospecimens will be retained as part of the study.

Psychological discomfort or distress: Some participants may experience emotional distress during the assessments, since measures address sensitive topics such as stigma, medical mistrust, and potential exposures to COVID-19. In our experience with COVID-19, however, these events are rare and almost all cases are short-lived and of low intensity. Further, since all participants will be linked to a PMHC site where mental health crises are routinely managed, all participants will be provided access to mental health care in the unusual instances of extreme emotional distress induced by COVID-19 testing.

Possibility of or perceived invasion of privacy to the participant or their family. Some participants may find the questions in this study to be invasive. Study measures will ask about social networks and relationships, including potential exposures to COVID-19 to close contacts, such as family members and friends. However, questions about COVID-19 exposure are based on standard of care and routine contact tracing. Furthermore, the overall magnitude of this risk is likely low, and participants can decline to answer any questions.

9.3 For each risk selected above, describe how you will manage or minimize the risk.

Breach of confidentiality: As described above, we have taken numerous measures to minimize breaches in confidentiality and to maintain confidentiality of our data. All research staff are trained in human subjects research. In the unexpected case of concern about breach of confidentiality, it will be reported directly to our Human Subjects Unit and the study PI to determine if it is substantiated. If so, the trial will be paused, a course of action to mitigate the disclosure will be effected and additional measures to prevent additional occurrences will be implemented prior to restarting the trial.

Psychological discomfort or distress: All of our study materials will include a study hotline that will allow participants to communicate any discomfort or distress occurring during the study. Study personnel, who have significant experience working with our target patient population will manage such occurrences should they occur.

Possibility of or perceived invasion of privacy to the participant or their family: Participants can skip or decline to answer all survey questions. Interviewers will also offer to conduct surveys at a different time if participants feel privacy cannot be ensured. As described above, personal information will be kept separate from study information.

For protocols that meet the criteria for exempt research, skip to Section 10.

9.4 Are there any risks to individuals related to pregnancy, fertility, lactation, or effects on a fetus or neonate? If yes, describe how you will manage or reduce this risk.

Some studies will require unique considerations if a participant becomes pregnant during the study (e.g., discontinuation of a diet-based intervention or medications).

There are no risks to individuals related to pregnancy, fertility, lactation, or effects on a fetus or neonate.

9.5 Describe whether participants who become pregnant will be able to continue their participation in the study. If they will continue to be included in the study, describe any measures to accommodate the pregnancy, if applicable.

Participants who become pregnant will be able to continue their participation in the study. We do not anticipate any effect of the study procedures on pregnancy.

9.6 Describe the potential direct benefits to individual participants or to others as a result of study participation. Indicate if there may not be any direct benefits to participants or to others.

There may not be direct benefits to Index Participants who enroll in the study. Network Contacts who are reached through the study will benefit from access to COVID-19 testing, and those in the self-testing arm will receive home test kits. All participants who enroll in this study, as well as individuals reached through the study through test referrals and self-testing kits, are offered COVID-19 testing and will have the opportunity to obtain further clinical care, including linkage to primary care, through PHMC.

9.7 Define reportable and non-reportable Adverse Events (AEs).

Reportable AEs:
We will not report AEs since COVID-19 self-testing is standard and the study cannot cause adverse effects from testing procedures.

Non-reportable AEs:
None.

9.8 Define reportable Serious Adverse Events (SAEs). Choose one or both as applicable to your study.

☐ Any adverse event that:

1. Results in death;
2. Is life-threatening (places the subject at immediate risk of death from the event as it occurred);
3. Results in inpatient hospitalization or prolongation of existing hospitalization;
4. Results in a persistent or significant disability/incapacity;
5. Results in a congenital anomaly/birth defect; or
6. Based upon appropriate medical judgment, may jeopardize the subject's health and may require medical or surgical intervention to prevent one of the other outcomes listed above.

(*OHRP Guidance on Unanticipated Problems and Adverse Events; January 15, 2007)*

☒ Other, or in addition to the above (*please describe*):
COVID-19 is known to cause severe illness and death. These events will not be tracked by the study since our goal is to increase the ability to diagnose the illness. It is implausible that our study procedures might result in an excess amount of morbidity and mortality from COVID-19 since testing is considered one of the key public health strategies to decrease these adverse effects. Our reporting will only be focused on any events we become aware of that could have resulted from our study procedures (e.g., intimate partner violence due to receiving a positive COVID test result).

9.9 Describe plans to identify, monitor, manage, and report AEs and SAEs. Include information on the method and frequency of collecting AE and SAE information (e.g., weekly through telephone calls, monthly at study visits, every time the subject comes to the clinical setting), the individuals responsible for collecting this information (e.g., research assistants, case managers, counselors), and the start and end of collection (e.g., from the participant's first exposure to the intervention to their last study visit).

Monitoring for Adverse Events (AE) will be conducted in real-time by the study personnel and the on-site study physician (Acri) and co-investigator (Dugosh). The study team will complete adverse effect assessments and will

determine the severity of the adverse events. The DSMB will make the determination of social, economic, and medical adverse events. The relationship of the adverse event to participation in the study will be classified as: definitely related, probably related, possibly related, unlikely or unrelated.

Subjects will also be given a study hotline they can call if necessary. The PI, Trial Director and on-site study physician will follow all subjects who are discontinued due to a serious adverse event from study procedures until it resolves and becomes completely stable. All study related SAEs will be documented on an Adverse Events Case Report Form and reported to the Human Subjects Committee within 48 hours.

All serious adverse events, as defined in the SAE procedures, will be reported within 24 hours to senior study personnel. These events will be maintained in a unique data base and reviewed monthly by senior study personnel. The site investigators will review all Serious Adverse Event forms in "real-time" to ensure appropriateness of the data and timeliness of reporting.

Staff training will consist of an explanation of the protocol and review of the Case Report Forms. In addition, the duties of each staff person will be outlined and all applicable regulations will be reviewed. Mock sessions with critical feedback will be conducted. Senior personnel will supervise junior staff and provide re-training in the study protocol as needed.

Dr. Dugosh and study research interviewers will be responsible for monitoring data integrity as data are collected. This includes ensuring that source documents exist for the data on the case report forms, ensuring all fields are completed appropriately, all corrections are done according to GCPs and any inconsistencies/deviations are documented.

The study will be monitored by the PI and co-investigators, and regulatory committees at PHMC and Penn (i.e., IRBs) as well as by a study specific DSMB.

9.10 Will you submit or have you submitted the protocol for additional oversight by a Data and Safety Monitoring Board (DSMB)?

☐ Yes:
- ☐ The protocol will be submitted to PHMC's DSMB.
- ☐ PHMC's DSMB conducted the initial review on .
- ☒ The protocol will be submitted to an external DSMB.
- ☐ An external DSMB or monitoring committee has reviewed the study. All reports are attached.

☐ No, a DSMB is not needed for this study

9. REFERENCES

1. Altamirano J, Govindarajan P, Blomkalns AL, et al. Assessment of Sensitivity and Specificity of Patient-Collected Lower Nasal Specimens for Sudden Acute Respiratory Syndrome Coronavirus 2 Testing. *JAMA Network Open.* 2020;3(6):e2012005-e2012005.
2. Johnson CC, Kennedy C, Fonner V, et al. Examining the effects of HIV self-testing compared to standard HIV testing services: a systematic review and meta-analysis. *J Int AIDS Soc.* 2017;20(1):21594.

3. Qin Y, Han L, Babbitt A, et al. Experiences using and organizing HIV self-testing. *Aids.* 2018;32(3):371-381.
4. Thirumurthy H, Masters SH, Mavedzenge SN, Maman S, Omanga E, Agot K. Promoting male partner HIV testing and safer sexual decision making through secondary distribution of self-tests by HIV-negative female sex workers and women receiving antenatal and post-partum care in Kenya: a cohort study. *Lancet HIV.* 2016;3(6):e266-274.
5. Giguere R, Lopez-Rios J, Frasca T, et al. Use of HIV Self-Testing Kits to Screen Clients Among Transgender Female Sex Workers in New York and Puerto Rico. *AIDS and behavior.* 2020;24(2):506-515.
6. Pal K, Ngin C, Tuot S, et al. Acceptability Study on HIV Self-Testing among Transgender Women, Men who Have Sex with Men, and Female Entertainment Workers in Cambodia: A Qualitative Analysis. *PloS one.* 2016;11(11):e0166129.
7. MacGowan RJ, Chavez PR, Borkowf CB, et al. Effect of Internet-Distributed HIV Self-tests on HIV Diagnosis and Behavioral Outcomes in Men Who Have Sex With Men: A Randomized Clinical Trial. *JAMA internal medicine.* 2019;180(1):117-125.
8. Lightfoot MA, Campbell CK, Moss N, et al. Using a Social Network Strategy to Distribute HIV Self-Test Kits to African American and Latino MSM. *J Acquir Immune Defic Syndr.* 2018;79(1):38-45.
9. Golden MR, Kerani RP, Stenger M, et al. Uptake and population-level impact of expedited partner therapy (EPT) on Chlamydia trachomatis and Neisseria gonorrhoeae: the Washington State community-level randomized trial of EPT. *PLoS Med.* 2015;12(1):e1001777.
10. Logie CH. Lessons learned from HIV can inform our approach to COVID-19 stigma. *J Int AIDS Soc.* 2020;23(5):e25504.
11. Sotgiu G, Dobler CC. Social stigma in the time of coronavirus disease 2019. *Eur Respir J.* 2020;56(2).
12. Bagcchi S. Stigma during the COVID-19 pandemic. *Lancet Infect Dis.* 2020;20(7):782.
13. He X, Liu G, Xia D, et al. An innovative HIV testing service using the internet: Anonymous urine delivery testing service at drugstores in Beijing, China. *PLoS One.* 2018;13(2):e0192255.
14. MacGowan RJ, Chavez PR, Borkowf CB, et al. Effect of Internet-Distributed HIV Self-tests on HIV Diagnosis and Behavioral Outcomes in Men Who Have Sex With Men: A Randomized Clinical Trial. *JAMA Intern Med.* 2020;180(1):117-125.
15. Vaughan D, O'Connell E, Cormican M, et al. "Pee-in-a-Pot": acceptability and uptake of on-site chlamydia screening in a student population in the Republic of Ireland. *BMC Infect Dis.* 2010;10:325.
