# Supplementary material for: Provision of COVID-19 Self-Test Kits to Patients for Distribution to Social Contacts: A Randomized Clinical Trial
Source: JAMA Netw Open. 2025 Jun 4;8(6):e2513708. doi: 10.1001/jamanetworkopen.2025.13708 (PMC12138724; doi:10.1001/jamanetworkopen.2025.13708)
Supplement: Supplement 2. — eTable 1. Characteristics of Participants at Baseline and 8-Week Follow-Up eTable 2. Number of Test Kits or Network Contacts Reached at 8-Week Follow-Up (n = 275) [file jamanetwopen-e2513708-s002.pdf]

Supplemental Online Content

Bien-Gund CH, Stephens-Shields AJ, Acri T, Dugosh K, Gross R. Provision of COVID-19 self-test kits to patients for distribution to social contacts: a randomized clinical trial. *JAMA Netw Open*. 2025;8(6):e2513708. doi:10.1001/jamanetworkopen.2025.13708

eTable 1. Characteristics of Participants at Baseline and 8-Week Follow-Up
eTable 2. Number of Test Kits or Network Contacts Reached at 8-Week Follow-Up (n = 275)

This supplemental material has been provided by the authors to give readers additional information about their work.

© 2025 Bien-Gund CH et al. *JAMA Network Open*.

eTable 1: Characteristics of participants at baseline and 8-week follow-up

		Total (N=776)	Completed 8-week follow-up (N=560)	Did not complete follow-up (N=216)	P-value
Age (Years)	Median (IQR)	44.0 (32.0, 57.0)	43.0 (32.0, 56.0)	46.0 (33.0, 60.0)	0.13
Race Hispanic	Black, not Hispanic or Latine	112 (14.4%)	87 (15.5%)	25 (11.6%)	0.30
	White, not Hispanic or Latine	459 (59.1%)	332 (59.3%)	127 (58.8%)	
	Hispanic or Latine, any race	120 (15.5%)	88 (15.7%)	32 (14.8%)	
	Asian/Pacific Islander, not Hispanic or Latine	21 (2.7%)	15 (2.7%)	6 (2.8%)	
	Multi-racial/Other, not Hispanic or Latine	64 (8.2%)	38 (6.8%)	26 (12.0%)	
Sex and gender identity	Cis-gender male	328 (42.3%)	216 (38.6%)	112 (51.9%)	0.001
	Cis-gender female	428 (55.2%)	325 (58.0%)	103 (47.7%)	
	Transgender/non-binary	20 (2.6%)	19 (3.4%)	1 (0.5%)	
Test location	Site 1	1 (0.1%)	0(0%)	1 (0.5%)	0.08
	Site 2	429 (55.3%)	298 (53.2%)	131 (60.6%)	
	Site 3	128 (16.5%)	94 (16.8%)	34 (15.7%)	
	Site 4	218 (28.1%)	168 (30.0%)	50 (23.1%)	
People lived with	Lives alone	184 (23.7%)	132 (23.6%)	52 (24.1%)	<.0001
	1 other person	167 (21.5%)	122 (21.8%)	45 (20.8%)	
	2-4 people	252 (32.5%)	199 (35.5%)	53 (24.5%)	
	5-10 people	61 (7.9%)	43 (7.7%)	18 (8.3%)	
	>10 people	50 (6.4%)	35 (6.3%)	15 (6.9%)	
	Unknown	62 (8.0%)	29 (5.2%)	33 (15.3%)	
Homeless	Yes	77 (9.9%)	48 (8.6%)	29 (13.4%)	
Education	Less than high school	113 (14.6%)	79 (14.1%)	34 (15.7%)	<.0001
	Graduated high school	272 (35.1%)	186 (33.2%)	86 (39.8%)	
	Some college or more	362 (46.6%)	282 (50.4%)	80 (37.0%)	
	Unknown	29 (3.7%)	13 (2.3%)	16 (7.4%)	
Annual household income	Less than $15,000	201 (25.9%)	131 (23.4%)	70 (32.4%)	0.007
	$15,000 to $49,999	272 (35.1%)	212 (37.9%)	60 (27.8%)	
	$50,000 to $74,999	84 (10.8%)	68 (12.1%)	16 (7.4%)	
	$75,000 or more	56 (7.2%)	45 (8.0%)	11 (5.1%)	
	Unknown	115 (14.8%)	82 (14.6%)	33 (15.3%)	

© 2025 Bien-Gund CH et al. *JAMA Network Open*.

		Total (N=776)	Completed 8-week follow-up (N=560)	Did not complete follow-up (N=216)	P-value
Employment status	Employed	386 (49.7%)	304 (54.3%)	82 (38.0%)	<.0001
	Unemployed	92 (11.9%)	61 (10.9%)	31 (14.4%)	
	Retired	54 (7.0%)	40 (7.1%)	14 (6.5%)	
	Disabled	128 (16.5%)	87 (15.5%)	41 (19.0%)	
	Student	35 (4.5%)	22 (3.9%)	13 (6.0%)	
	Other/unknown	81 (10.4%)	46 (8.2%)	7 (16.2%)	

P-values are based on chi-squared test for categorical variables, and Wilcoxon rank sum test for continuous variables.

© 2025 Bien-Gund CH et al. *JAMA Network Open*.

eTable 2: Number of test kits or network contacts reached at 8-week follow-up (N=275)

	Number of test kits or network contacts reached (%)					
	**0**	**1**	**2**	**3**	**4**	**5**
Self-tests given out	31 (11.5)	16 (5.9)	30 (11.1)	52 (19.3)	52 (19.3)	89 (33.0)
Network contacts reached	37 (13.5)	26 (9.5)	43 (15.6)	54 (19.6)	41 (14.9)	74 (26.9)
Use of test kit on self	211 (78.1)	42 (15.6)	10 (3.7)	3 (1.1)	1 (0.4)	3 (1.1)
Unused test kits in possession	130 (47.3)	47 (17.1)	43 (15.9)	21 (7.6)	16 (5.9)	15 (5.6)

Rows may not sum to total N due to missing data.

© 2025 Bien-Gund CH et al. *JAMA Network Open*.
