# Supplementary material for: Provision of COVID-19 Self-Test Kits to Patients for Distribution to Social Contacts: A Randomized Clinical Trial
Source: JAMA Netw Open. 2025 Jun 4;8(6):e2513708. doi: 10.1001/jamanetworkopen.2025.13708 (PMC12138724; doi:10.1001/jamanetworkopen.2025.13708)
Supplement: Supplement 3. — Data Sharing Statement [file jamanetwopen-e2513708-s003.pdf]

Data Sharing Statement

Bien-Gund. Provision of COVID-19 Self-Test Kits to Patients for Distribution to Social Contacts. *JAMA Netw Open*. Published June 04, 2025. doi:10.1001/jamanetworkopen.2025.13708

Data
Additional Information: Clinicaltrials.gov NCT: 04797858
Data available: Yes
Data types: Deidentified participant data
How to access data: Data will be made available upon request.
When available: With publication

Supporting Documents
Document types: None

Additional Information
Who can access the data: Data will be made available upon request whose proposed use of the data has been approved.
Types of analyses: Secondary analyses whose proposed use of the data has been approved.
Mechanisms of data availability: A signed data access agreement must be signed prior to data becoming available.
